# Bullying and Victimization among Students Bears Relationship with Gender and Emotional and Behavioral Problems

**Published:** 2019-07

**Authors:** Maryam Mohseny, Zahra Zamani, Shahin Akhondzadeh Basti, Mohammad-Reza Sohrabi, Ali Najafi, Jayran Zebardast, Farzad Tajdini

**Affiliations:** 1 Department of Community Medicine, Shahid Beheshti University of Medical Sciences, Tehran, Iran.; 2 Psychiatric Research Center, Roozbeh Hospital, Tehran University of Medical Sciences, Tehran, Iran.; 3 Department of Medical Library & Information Sciences, Tehran University of Medical Sciences, Tehran, Iran.; 4 Department of Cognitive Sciences Special Linguistics, Institute of Cognitive Sciences University, Tehran, Iran.

**Keywords:** *Adolescent*, *Bullying*, *Prevalence*, *School of Health*, *Victimization*

## Abstract

**Objective:** Bullying and victimization are common and serious problems in schools resulting in development of emotional and behavioral disorders in adolescents. This study aimed at examining the prevalence of bullying behavior and some of its associated factors among students.

**Method** **:** This was a cross sectional analytic study involving junior high schoolers in grades seven, eight and nine. This study was part of an international study that used a questionnaire as a tool for data collection. The questionnaire investigated some of the characteristics and qualities possessed by most juveniles and some occasional problems which they may experience. Also, it was used to examine participants’ experiences with bullying and victimization. This questionnaire comprised of 15 sections on demographic characteristics, individual health, family status and types of bullying experiences at school and outside of school, along with the Strengths and Difficulties Questionnaire (SDQ), which is an instrument for screening emotional and behavioral problems in children and adolescents. A multistage cluster sampling from five regions, consisting of the north, south, west, east, and central regions of Tehran, was conducted and 1456 questionnaires were completed by the students.

**Results: **According to the results of this study, prevalence rate for bullying and victimization was 17.4% and 25.8%, respectively. The results indicated that gender had a significant relationship with bullying and victimization, with boys being more likely to be bullies and also more prone to victimization than girls (p < 0.001). Other parameters such as emotional, behavioral, and environmental influence also had a significant relationship with bullying and victimization.

**Conclusion: **Bullying is more prevalent in boys than in girls, and boys are more likely to be victimized as well. Emotional and behavioral problems are identified as risk factors, and future interventions should focus on these risk factors to develop preventive measures.

Bullying is defined as a proactive form of aggression which is intentional, repeated over time, and characterized by a power imbalance between the perpetrator and the victim (1). There are three different key players involved in bullying: the bully, the victim, and the bully-victim. Bullying has been classified by two modes and three types. Modes of bullying are direct and indirect and types of bullying are physical, such as hitting and kicking, verbal, such as name-calling, taunting, and threatening, and psychological (social exclusion, isolation, and malicious gossip) (2,3). Bullying behaviors are found in both genders in all racial and ethnic groups, in different cultures, and in all socioeconomic groups. Negative impacts of bullying are felt by individuals, families, schools, and the society.

Thus, it is an important public health issue that has gained worldwide attention in the past decade (4). 

Dan Olweus was the first person who paid attention to the bullying phenomenon in the late 1970s (1). Results of an international research on bullying and victimization have shown a wide range of difference around the world from 12% in Australia to 83% in Saudi Arabia [5, 6]. In a recent systematic review conducted in Iran, prevalence of bullying was 30% to 65% (7). Different studies have identified several factors associated with bullying such as gender, age, physical appearance, individual tendency, and family factors, including marital conflict, domestic violence, child abuse, and low income or unemployment (8–10). 

Several studies have reported a relationship between bullying and health problems in school children. Bullying and victimization lead to different types of physical and psychological health problems, including nausea, headache, vomiting, abdominal pain, bed-wetting, and increased or decreased appetite. It has been reported that bullies are more likely to experience social psychological adjustment problems such as depression, loneliness, and social isolation (11). The potential consequences for victims include loss of sleep, depression, lower motivation to attend school, psychosomatic problems, low self-esteem or self-worth, and even suicide and suicidal tendencies. A good number of investigations suggest that peer victimization is an important risk factor for adolescent suicide (12–14). Additionally, there is an association between bullying and substance use among high schoolers. Higher prevalence of cigarette smoking and alcohol use was observed in victims, compared to their non-bullied peers, due to experiencing such symptoms as depression and anxiety (15, 16).

A recent meta-analysis has identified 4 predictors of victimization (prior victimization, conduct problems, social problems, and internalizing problems) and also 4 predictors of bullying (conduct problems, social problems, school problems, and age) (17). 

Although some research has been done on bullying in Iran, far too little attention has been paid to bullying prevalence, its consequences, and related factors especially in adolescents. This study was the first to show a relationship between bullying and physical and mental health based on a detailed questionnaire, which was a part of an international comparative study in Finland representing an ongoing European and Asian interstate investigation. The recent exacerbation of violence and bullying among the adolescents has become a serious public health problem and extensive studies are necessary in this regard.

The present study had two primary aims: (1) to evaluate the prevalence of bullying and victimization, and (2) to investigate any relationship between bullying and victimization experienced by junior high schoolers based on the mentioned questionnaire.

## Materials and Methods

***Participants & Sampling***

This was a cross sectional analytic study for which a multistage clusters sampling method was used and the survey included male and female students of both governmental and non-governmental institutions. Using the sample size calculation, 1456 high schoolers in grades seven, eight and nine from north, south, west, east, and central regions of Tehran were selected to participate in this study and fill out the questionnaires.

***Ethics***

Approval for this study was granted by the ethics committee of Tehran University of Medical 

Sciences (1394.1413). 

***Study Design***

Anonymous printed questionnaires were distributed and guided by four experienced questioners (two men for boys and two women for girls’ schools) who were trained to administer the questionnaire in a consistent manner. At first, informed consent was obtained from all students. Students were assured of the confidentiality of their questionnaire responses. An average of 20 minutes was required to complete the questionnaires, and a total of 20 institutions were covered within six months in the five mentioned regions. 

***Study Protocols & Instrument***

This survey was part of an international comparative study in Finland, representing an ongoing European and Asian interstate investigation. Whereas previous studies have mostly used the Olweus questionnaire, in this study, a comprehensive questionnaire was designed for use at international level, with 15 sections: (1) demographic, (2) family status, (3) background, (4) individual health, (5) aches & sleeps, (6) thoughts about body, (7) substance use, (8) Strengths and Difficulties Questionnaire (SDQ), (9) experience about difficulties, (10) suicidality, (11) need for outside help, (12) experience of being bullied, (13) cyberbullying (not being studied in this survey), 14) bullying in general (at school, outside school, on the internet), and (15) school environment. The following information was collected and analyzed: basic information about bullying behavior (traditional bullying), psychosomatic symptoms (headache, stomachache and sleep problems), concerns about safety at schools, help-seeking behaviors, questions about risky behaviors. Also, some of the characteristics and qualities possessed by most juveniles and some occasional problems that they might have experienced were investigated. 

The questionnaire was used by Ander Sourander, as the main designer of the project, for the first time in 2004 and was also used in the current international study (18). Cronbach's alpha was used for reliability of the questionnaire and was calculated to be 0.73. This questionnaire was prepared based on back-translation and the Persian version of the questionnaire was used in this study. The Strengths and Difficulties Questionnaire (SDQ) (section 8) is an instrument for screening emotional and behavioral problems in children and adolescents (19, 20). The concurrent validity and internal consistency of the SDQ was evaluated comparing the subscales of the SDQ by Tehrani-Doost et al in 2009 and was found to be good and strong correlations were found among similar subscales (21).

The SDQ, developed by Goodman, is an instrument for investigating mental health and behavioral problems in children and adolescents (22–26). This questionnaire was also used in previous Finland studies (27, 28). The reliability and validity of the SDQ (parent and self-report) were investigated in 1997 by Goodman et al in the UK (29, 30). 

SDQ is a 25-item behavioral screener and the score for each item in this structure ranges from 1 to 3 (1 = false, 3 = completely correct). In the present study, every 5 items were evaluated based on the instructions. Hence, the SDQ scales included social, hyperactivity, emotional, conduct and peer problems (23). Also, in this study, other variables such as school environment, body thought, and personal smoking and substance abuse were computed in relation to bullying and victimization (Table 3). After collecting the questionnaires, variable relationships with bullying and victimization were examined. 

This study was designed to be a part of the Finnish international European and Asian interstate study. 

***Statistical Analyses***

In this study, SPSS 16 software was used for statistical analysis. Descriptive and analytic statistics were used to present data distribution and to assess the association between different variables and bullying behavior. Chi-square test was used to obtain the association of demographic variables with bullying and victimization and victim or victim-bully groups. To assess the relationship between bullying behavior and quantitative variables, t test, Mann- Whitney test, and ANOVA were used. Logistic regression analysis was conducted to test a model predicting bullying behavior.

## Results

***Descriptive Findings***

A total of 1456 questionnaires were completed by the participants. Of the participants, 794 were male and 662 were female. Table 1 demonstrates the demographic characteristics of the participants. Of the participants, 54.5% were male and 45.5% were female, and most of whom were in 14-15 age group and in the 9th grade. Moreover, 88.8% of the participants studied in governmental schools; 93.6% lived with their biological parents. In a brief survey about parents' occupations in this study, it was found that most of the fathers were self-employed while most mothers were housewives.

**Figure 1:**
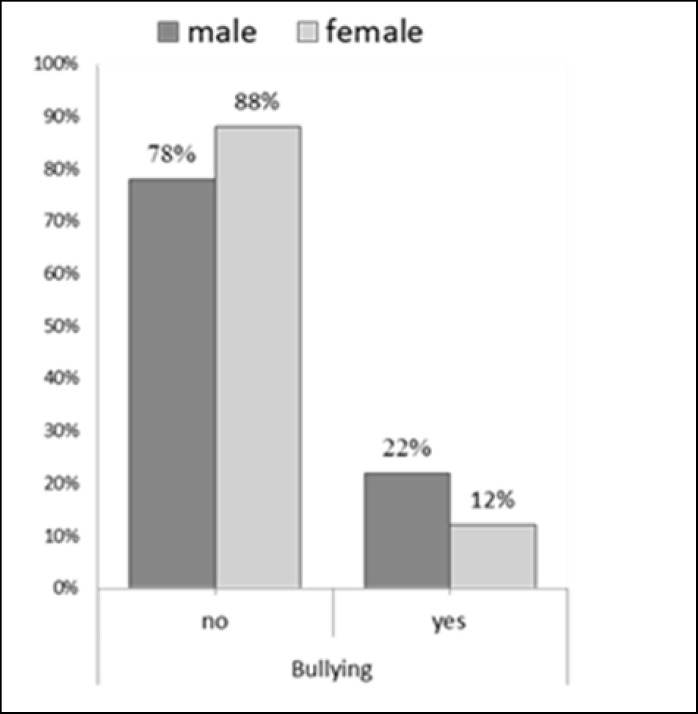
Frequency Status of Bullying between Male and Female Students

**Figure 2:**
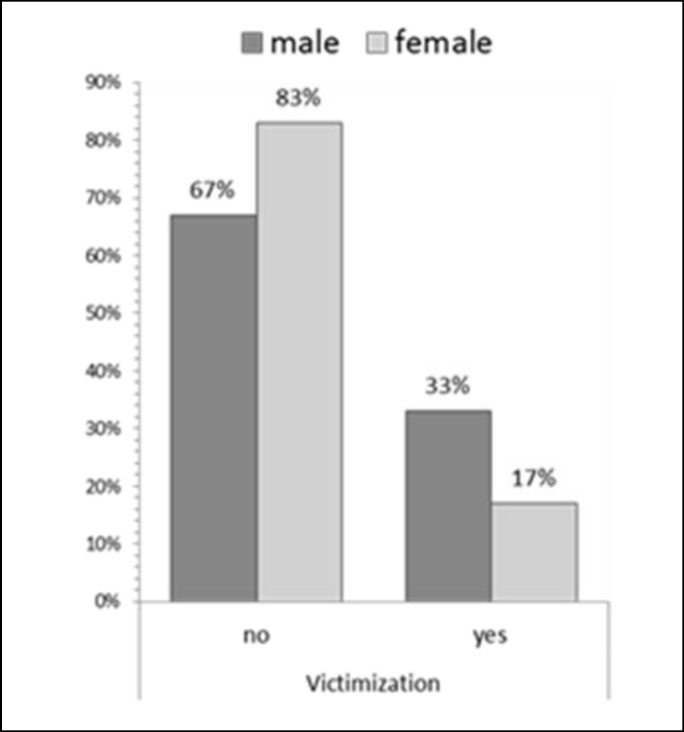
Frequency Status of Victimization between Male and Female Students

Overall, the results showed a prevalence of 17.4% for bullying, 25.7% for victimization, and 30.5% for both. Also, bullying and victimization were significantly higher in boys than in girls (p < 0.001) (Figure 1, 2).

***Analytical findings***

Table 2 presents the association between demographic characteristics of the participants with bullying and victimization. Both gender and high school type had a significant association with bullying and victimization (p < 0.01). Students in the 13-14 age group had the most rate of victimization, while the most rate of bullying was observed among the 15-16 age group than other age groups.

Table 3 shows a correlation between quantitative and computed variables with bullying and victimization. Significant correlations were found between non-victim & victim with weight (58.23 vs 60.31) (p = 0.04), peer problems (1.53 vs 1.68) (p < 0.001), conduct problems (1.50 vs 1.59) (p < 0.001), hyperactivity problems (187 vs 1.94) (p = 0.001), emotional problems (1.40 vs 1.58) (p < 0.001), body thought problems (2.22 vs 2.16) (p = 0.003), school environment problems (2.53 vs 2.39) (p < 0.001), and smoking and substance abuse (1.87 vs 1.78) (p = 0.02).

Height, weight, BMI, peer, conduct and hyperactivity, social, emotional, body thought, school environment, and smoking and substance abuse demonstrated significant correlations with bullying.

The mean of these variables in both non-bully & bully groups were as follow: height (165.5 vs 167.3) (p = 0.03), weight ( 58.1 vs 61.9) (p = 0.002), BMI (21.1vs 22.0) (p = 0.01), peer problems (1.55 vs 1.66) (p < 0.001), conduct problems (1.49 vs 1.67) (p < 0.001), hyperactivity(1.88 vs 1.95) (p < 0.001), social problems (1.82 vs 1.88) (p = 0.008), emotional problems (1.41 vs 1.62) (p < 0.001), body thought problems (2.21 vs 2.15) (p = 0.003), school environment problems (2.52 vs 2.35) (p < 0.001), and smoking and substance abuse (1.87 vs 1.71) (p = 0.001).

To determine the predictability of factors such as gender, need to help others, smoking and substance abuse, and hyperactivity associated to bullying and victimization, a logistic regression analysis was performed (95% confidence interval). The computed variables were all predictive for both bullying and victimization as indicated by their p-values (Tables 4 and 5). Gender, peer problems, school environment problems, and smoking and substance abuse were found to be predictors in both bullying and victimization.

## Discussion

The initial objective of this study was to investigate the prevalence of bullying and victimization involving a population of high schoolers in grade seven, eight and nine in Tehran. According to the findings of this study, the rate of bullying was 17.4% while that of victimization was 25.7%, and the general prevalence rate was 30.5% for both. This study also illustrated that gender significantly correlated with bullying behaviors despite the fact that the bully was more likely to be from the male gender. Boys were also more likely to be victimized as opposed to girls. 

In a systematic review on Iranian adolescents, the bullying incidence was rated between 30% and 65.5%, with males being 2.5 folds more affected than females (7). Several investigations performed in different geographical locations worldwide have exhibited a disparity in the incidence and prevalence of bullying status (bully only, victim only, and bully and victim). A recent survey conducted in the United States reported a 26% incidence rate, with 12% for bullying and 19% for victimization in Washington (12). Other studies recorded higher incidence rates in other regions such as 38% in Iran and 83% in Saudi Arabia (5, 31). These inconsistences may result from the employed survey methodology, cultural diversity, living environment, and the size and the density of the cities. The present study revealed that bullying is committed more by male adolescents who are also more victimized, which is consistent with some previous investigations (11, 17, 32 and 33). Contrary to the studies by Lara and also by Vieno, females are more victimized than males and this disparity may be due to cultural differences between the study populations, different educational levels (grade 9-11), and different study methodology (16, 34).

The second objective of this study was to investigate the relationship between bullying and victimization with demographic, physical, emotional, and behavioral characteristics. Based on findings of this study, tobacco and alcohol use have significant correlation with bullying and victimization. This should be considered in light of the fact that this was the first nationally representative study to explore the associations between bullying and triggering bullying behaviors such as tobacco and alcohol use in adolescents. Several studies have been conducted in other countries on the association between smoking or alcohol consumption in students and bullying behaviors and they confirm the results of the present study (35–37). These findings are expected because students with violent risk factors are more likely to be smokers and alcohol users and harbor bullying behaviors as well (38).

The most important finding in this study was the positive and significant correlation between conduct, emotional, social, peer and hyperactivity problems (SDQ items) with bullying behaviors although social problems had a significant correlation only with bullying (not victimization). A current meta-analysis (Kljakovic et al) conducted on predictors of bullying confirmed that conduct, social, peer and school problems predict bullying behaviors. In other surveys in Finland, guided by Sourander, these factors are corroborated as a risk factor for cyber bullying (10, 12, 17 and 39). 

The current study found that physical problems influence bullying behaviors, as Parker's study confirms this and suggests dermal illness such as atopic dermatitis causes stigma and those affected are prone to bullying behaviors. Findings by Antonella explained that food allergies and physical inability are related to bullying. Nevertheless, Minne Fekkes states that psychosomatic and psychological disorders are related to bullying behaviors but not physical illness (12, 40 and 41). 

According to findings of this study, BMI, height, and weight were significantly correlated with bullying but only weight had a significant relationship with victimization. This is contrary to Johnson’s study that found obese individuals were more likely to perpetrate bullying than their normal-weight classmates or Lara's study that showed being overweight is associated only with victimization (34, 42). 

According to previous studies, teacher-student relationship is highly important in prohibiting violence between students. In other words, students who have a more positive interaction with their teachers feel more confident, and feelings of friendship and intimacy with a teacher will reduce incidence of maladaptive bullying behaviors. They support the findings of the present study that demonstrated school environmental problems had a significant correlation with bullying and victimization (37, 43–45).

In this study, surprisingly, a significant difference was found in the rate of violence among students in public versus private high schools, finding more bullying and victimization in private high schools. However, there is scarcity of data regarding this issue. A possible explanation for these results may be lack of supervision and rigidity from teachers and school masters in private schools, and this makes students, who are prone to bullying behaviors, bully others easily. Thus, further studies with more focus on the relationship between school type and violence is suggested.

Moreover, this study illustrated a relevance between victimization and birthplace (Tehran or other cities). Those who were born in Tehran were less likely to be victims. Cultural diversity, parental income, higher self-confidence, and self-esteem can possibly prevent victimization and should be investigated in future surveys. Interestingly, there were also differences in rates of bullying and victimization between students who felt they need help from others and those who did not. It is likely that individual characteristics are more significant than environmental impacts on emergence of these types of behaviors. 

Since primary prevention of bullying and victimization seem to be important, it is suggested that the impact of the proposed strategies on the reduction of violent behaviors in schools be examined using an empirical study with an interventional method. Another suggestion is to conduct a study of cyberbullying or electronic bullying on students because of the increasing use of digital media such as e-mails, text messages, social networks, and cellphone which can increase cyberbullying. 

**Table 1:** Demographic Characteristics of the Students

**Demographic variables**	**Variables**	**Frequency**	**%**	**Demographic variables**	**Variables**	**Frequency**	**%**
Gender	Male	794	54.5	Mother’s occupation	doctor	17	1.3
	Female	662	45.5		engineer teacher	5	0.4
Age groups	12-13	261	17.9		teacher	55	4.2
	14-15	947	65		self-employed	74	5.6
	16-17	248	17		employee	119	9.1
High school grade	7th grade	367	25.2		house wife	1001	76.4
	8th grade	380	26.1		unemployed	7	0.5
	9th grade	709	48.7		retired	33	2.5
High school type	public	1293	88.8	Student background	Tehran origin	946	65
	private	163	11.2		other	510	35
Parents with whom I live	biological parents	1330	93.6	Place of birth	Tehran	1261	86.6
	biological father and foster mother	10	0.7		other	195	13.4
	biological mother and foster father	7	0.5	Native language	Persian	1409	96.8
	biological father alone	14	1		other language	47	3.2
	biological mother alone	55	3.9	Birthplace of mother	Tehran	834	57.3
	Adoptive child	5	0.4		other	621	42.7
Father’s occupation	doctor	13	1	Native language of mother	Persian	1293	88.8
	Engineer/ teacher	71	5.4		other	163	11.2
	teacher	20	1.5	Birthplace of father	Tehran	780	53.6
	self-employed	594	45		other	675	46.4
	employee	447	33.9	Native language of father	Persian	1266	87
	laborer	45	3.4				
	unemployed	15	1.1		other language	190	13
	retired	62	4.7				
	others	53	4				

**Table 2:** Association between Bullying and Victimization with Demographic Characteristics

**Demographic variables**	**Variables**	**Victim**					**Bullying**				
		**No**		**Yes**		**P-value**	**No**		**Yes**		**P-Value**
		**N**	**%**	**N**	**%**		**N**	**%**	**N**	**%**	
Age groups	12-13	195	75.3	64	24.7	0.32	223	86.1	36	13.9	0.25
	13-14	688	73.1	253	26.9		770	82.0	169	18.0	
	15-16	191	77.6	55	22.4		200	81.3	46	18.7	
Gender	Male	533	67.1	261	32.9	<0.001	620	78.1	174	21.9	<0.001
	Female	541	83.0	111	17.0		573	88.2	77	11.8	
Grade	7th grade	277	75.9	88	24.1	0.67	312	85.5	53	14.5	0.1
	8th grade	281	74.3	97	25.7		299	79.5	77	20.5	
	9th grade	516	73.4	187	26.6		582	82.8	121	17.2	
High school type	public	966	75.3	317	24.7	0.01	1071	83.6	210	16.4	0.008
	private	108	66.3	55	33.7		122	74.8	41	25.2	
My parents with whom I live	biological parents	994	75.3	326	24.7	0.06	1094	83.0	224	17.0	0.28
	Only father or mother	60	65.9	31	34.1		71	78.0	20	22.0	
Father’s job	doctor	11	84.6	2	15.4	0.61	12	92.3	1	7.7	0.44
	engineer	50	70.4	21	29.6		55	77.5	16	22.5	
	teacher	12	60.0	8	40.0		16	80.0	4	20.0	
	self-employed	438	74.2	152	25.8		486	82.5	103	17.5	
	employee	331	74.9	111	25.1		363	82.1	79	17.9	
	laborer	35	77.8	10	22.2		37	82.2	8	17.8	
	unemployed	13	86.7	2	13.3		10	66.7	5	33.3	
	retired	49	80.3	12	19.7		55	90.2	6	9.8	
	others	41	77.4	12	22.6		45	86.5	7	13.5	
Mather’s job	Doctor or engineer	17	77.3	5	22.7	0.47	20	90.9	2	9.1	0.46
	teacher	44	80.0	11	20.0		48	87.3	7	12.7	
	Self-employed	55	74.3	19	25.7		59	79.7	15	20.3	
	employee	97	82.2	21	17.8		102	87.2	15	12.8	
	house wife	729	73.4	264	26.6		811	81.8	181	18.2	
	retired	5	71.4	2	28.6		5	71.4	2	28.6	
	others	25	78.1	7	21.9		28	87.5	4	12.5	

**Table 3:** Association between SDQ and Other Variables with Bullying and Victimization Presented as Mean ± SD Including Their Corresponding P-values

**Computed variables**	**Victimization**					**Bullying**				
	**No**		**Yes**		**P-Value**	**No**		**Yes**		**P-Value**
	**Mean**	**SD**	**Mean**	**SD**		**Mean**	**SD**	**Mean**	**SD**	
**Age**	14.54	1.04	14.57	0.95	0.5	14.52	1.03	14.65	0.97	0.06
**Height**	165.67	9.30	166.38	10.44	0.3	165.55	9.39	167.30	10.50	0.03
**Weight**	58.23	13.68	60.31	15.93	0.04	58.11	13.82	61.99	16.17	0.002
**BMI**	21.19	4.19	21.68	4.75	0.1	21.16	4.22	22.09	4.81	0.01
**Peer problems**	1.53	0.35	1.68	0.40	<0.001	1.55	0.37	1.66	0.37	<0.001
**Conduct problems**	1.50	0.35	1.59	0.39	<0.001	1.49	0.34	1.67	0.40	<0.001
**Hyperactivity problems**	1.87	0.26	1.94	0.29	0.001	1.88	0.27	1.95	0.28	<0.001
**social problems**	1.82	0.33	1.86	0.34	0.063	1.82	0.34	1.88	0.33	0.008
**Emotional problems**	1.40	0.42	1.58	0.48	<0.001	1.41	0.42	1.62	0.48	<0.001
**Body thought problems**	2.22	0.29	2.16	0.29	0.003	2.21	0.29	2.15	0.29	0.003
**School environment problems**	2.53	0.61	2.39	0.53	<0.001	2.52	0.59	2.35	0.58	<0.001
**Smoking and substance abuse**	1.87	0.33	1.78	0.41	0.02	1.87	0.32	1.71	0.45	0.001

SDQ: Strengths and Difficulties Questionnaire

**Table 4:** Logistic Regression Analysis of Victimization Predictors among Students

**Victimization predictors**		**Victimization**		
		**OR**	**CI **_95%_	**P-Value**
**Gender**	Male	4.65	2.76-7.84	<0.001
	Female	Reference		
**Peer problems**		2.27	1.5-4.5	0.02
**Emotional problems**		2.20	1.44-5.78	0.007
**Smoking and substance abuse**		2.25	1.77-3.3	0.02
**Conduct problems**		2.75	1.22-4.75	0.004
**Body thought problems**		3.05	1.13-4.5	0.03
**School environment problems**		2.75	1.27-5.95	0.001

**Table 5:** Logistic Regression Analysis of Bullying Predictors among Students

**Bullying predictors**		**Bullying**		
		**OR**	**CI **_95__%_	**P-Value**
**Gender**	Male	3.1	1.85-6.10	<0.001
	Female	Reference		
**Smoking and substance abuse**	Yes	2.21	1.10-4.17	0.01
	No	Reference		
**Hyperactivity**		3.02	1.10-8.07	0.01
**School environment problems**		0.88	0.18-1.05	0.001
**Peer problems**		0.70	0.59-2.25	0.002

## Limitation

A few limitations should be noted in this study. Firstly, due to the cross sectional nature of this investigation, the results cannot be used to establish any cause and effect interactions. Hence, to understand the causal relationships among variables, longitudinal designs are recommended. Secondly, participants for this study were only drawn from Tehran and the results may not be representative of the students in other regions of the country. Therefore, in this study, it was not possible to directly compare bullying and victimization among students in different cities. Thirdly, in this study, only junior high school students were included (grades 7–9). Since bullying is common in all ages, investigations of other grades seems necessary. Thus, bullying should be investigated in other school grades in future studies. The relatively small sample size of this study can be another limitation in this survey, meaning that study findings need to be interpreted with caution.

## Conclusion

The results of this study demonstrated that gender plays a crucial role in bullying behaviors. Bullying is more prevalent in boys compared to girls and boys are more likely to be victimized. Emotional, social, conduct, peer, and hyperactivity problems have a significant correlation with bullying and victimization. There is a relationship between demographic, physical, emotional, and behavioral characteristics with bullying and victimization. Tobacco and alcohol use have a significant correlation with bullying. Efforts should continue to identify how bullying behaviors can be prevented and intervention programs can be instituted.
